# The NetSage measurement and analysis framework in practice

**DOI:** 10.1007/s10586-021-03417-x

**Published:** 2021-11-06

**Authors:** Jennifer M. Schopf, Katrina Turner, Dan Doyle, Andrew Lake, Jason Leigh, Brian L. Tierney

**Affiliations:** 1 Indiana University, Bloomington, Indiana USA; 2 University of Hawaii, Honolulu, Hawaii USA; 3 Lawrence Berkeley National Laboratory, Berkeley, California USA

**Keywords:** Network measurement and monitoring, Visualization, Analytics, R&E Networks

## Abstract

Data sharing is required for research collaborations, but effective data transfer performance continues to be difficult to achieve. The NetSage Measurement and Analysis Framework can assist in understanding research data movement. It collects a broad set of monitoring data and builds performance Dashboards to visualize the data. Each Dashboard is specifically designed to address a well-defined analysis need of the stakeholders. This paper describes the design methodology, the resulting architecture, the development approach and lessons learned, and a set of discoveries that NetSage Dashboards made possible.

## Introduction

Scientific investigation is highly collaborative and requires the ability to seamlessly share data between institutions to enable scientific discovery. However, effective data sharing, especially for large data sets, can be challenging. For example, a common astronomy workflow involves a telescope producing data sets of 100 TBytes of data every day, which are then sent to multiple international sites for analysis. The data transfers and individual site processing must complete before the next data collection window in order to re-calibrate the telescope. If the data is not received in a timely fashion, the telescope cannot get re-focused and a viewing window may be lost. In another example, it took over three months to transfer data from a set of climate science experiments to a central location for analysis [16]. It is not uncommon for researchers to resort to shipping disks instead of using the network for data delivery due to network performance issues, such as is done for the Event Horizon Telescope black hole analysis [19]. Its also not unusual for a research collaboration to poorly estimate the time it may take to transfer a data file, such as when the Arecibo Telescope data center planned to back their data up in the cloud, and assumed it would take weeks and not the multiple years required by their initial approach.

The ability to measure and interpret network behavior is critical to understanding data transfer performance. Information about the end-to-end data path makes it possible to identify and address problems that are found. Our experience has shown that without this data, researchers often misunderstand the true performance of their data transfers, which can significantly impact their workflows and time to science.

This paper details the NetSage Measurement and Analysis Framework [56, 81], which is used to understand data transfer performance. We describe the stakeholder-focused methodology used to design performance Dashboards, each of which is focused on specific user questions. We detail the software architecture and its implementation that uses multiple data sources and takes advantage of related approaches. We then walk through several use cases to show the types of analyses and discoveries that the NetSage Framework enables.

This paper describes both the international and US domestic deployments of NetSage Portals as of April 2021. Previous publications about the NetSage Framework, for example [93], only addressed the International Portal and a minimal set of components and use cases. Other publications, such as [29, 30], focused on how the project worked with large-scale data and some very preliminary deployments prior to wide-scale collection of flow data. Presentations by the NetSage Project at numerous venues are available on the NetSage Project Website [56].

## NetSage overview

The NetSage Framework was created around a set of three design principles:

- NetSage *unifies* a broad set of data into a cohesive visualization, thereby enabling additional discoveries not possible with only a single data source.
- NetSage is *open* in that the data sets collected are meant to be widely accessible, with performance Dashboards open to the public. No other monitoring approach shares as much data in a public setting. None of the data shared is considered private or public, but making it widely available increases the usability and the audience. The NetSage is also available under open source license and available via GitHub [52].
- NetSage is *privacy-aware* and was developed with privacy concerns in mind. It contains no personally identifiable information (PII) about individual hosts or users of the network. Information about small flows, such as common with email or web searches, is also discarded. Also, if required by the data provider, the Dashboards can be secured by password or Shibboleth [42]. *The Dashboards described in this paper are all open to the public. We encourage readers to visit the URLs cited to see the full Dashboards in detail.*

The innovative aspect of NetSage is not in the individual pieces but rather in the integration of data sources to support objective performance observations as a whole. We refer to the *NetSage Framework* as an inclusive term for the methodology, architecture, and approach. *NetSage deployments* can collect data from routers, switches, active testing sites, and science data archives. A NetSage deployment uses a combination of passive and active measurements to provide longitudinal performance visualizations via *performance Dashboards*. We refer to a set of performance Dashboards for a project or set of resources as the *NetSage Portal* for that project or set of resources. The Dashboards can be viewed by resource collection, institutions or projects to identify changes of behaviors for data transfers using visualizations of data over time periods, as described in Sect. 6.

The NetSage Framework was developed for use by a broad user community, not by a single Network Operations Center (NOC). It was developed not only for network engineers, but resource owners, research technology departments, application scientists, CIOs, and funding agencies. The design approach was to meet a set of end user questions through innovative Dashboards, not just to supply measurement data to a NOC, which is the primary use case for most of the projects cited in the Related Work section.

The main use cases for the NetSage Framework have included:

- Understand the data movement patterns across a suite of resources;
- Identify the main sources and destinations for large data transfers, or flows;
- Visualize information about different research projects and science domains that are moving data;
- Display patterns of behaviors for data movement between organizations.

**Fig. 1:**
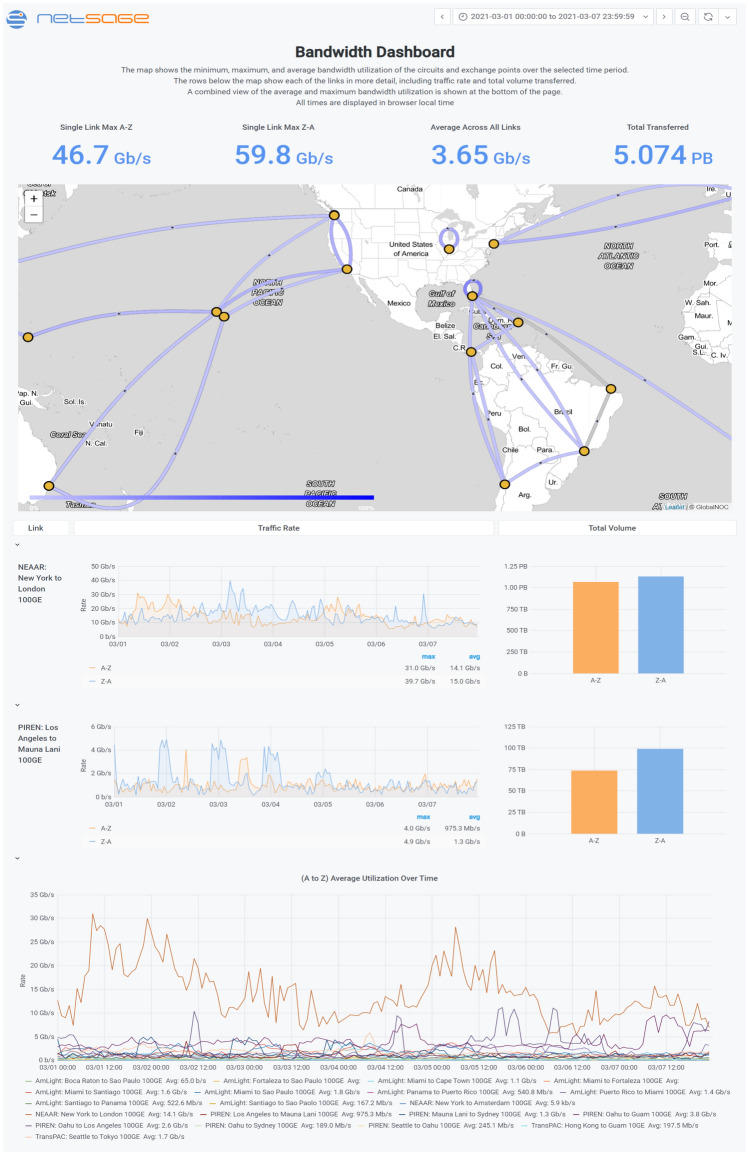

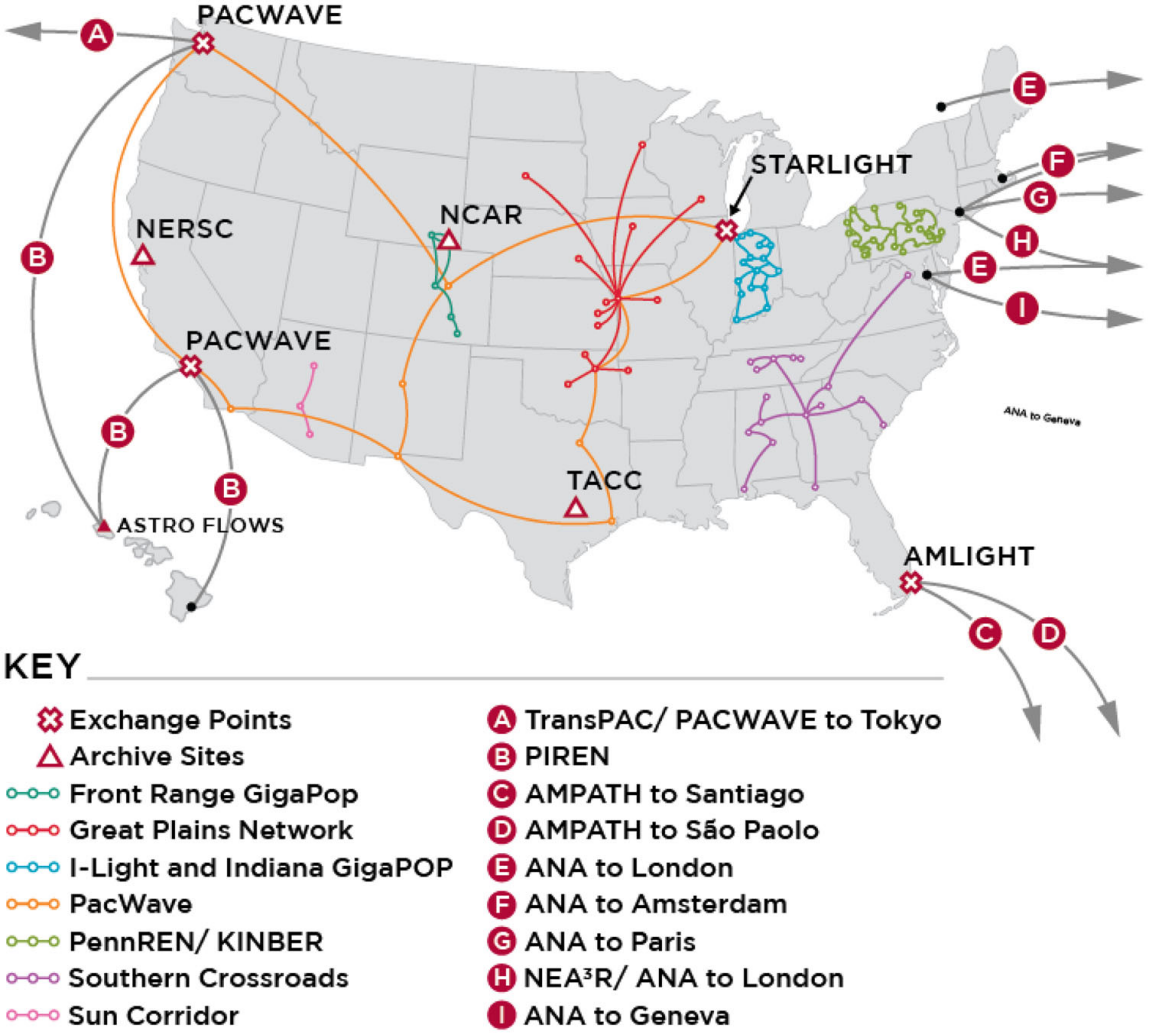
A map showing the US domestic and international deployments for NetSage as of March 2021

**Fig. 2:**
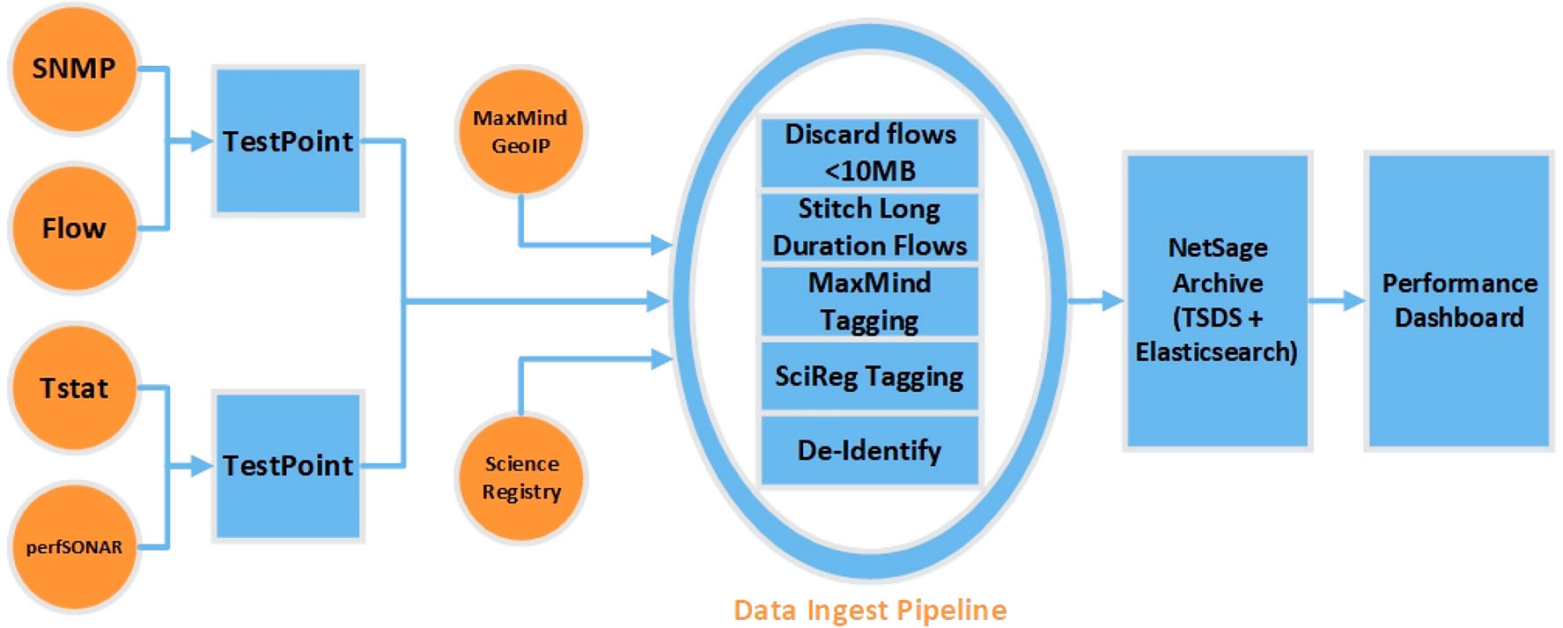
A logical diagram of the NetSage Architecture, consisting of Data sources, the Data Ingest Pipeline, the NetSage Archive, and sets of performance Dashboards

NetSage deployments now encompasses 14 regional deployments, listed in Table 1 and shown in Fig.  1. The NetSage Project was originally funded as part of the National Science Foundation (NSF) International Research and education Network Connections (IRNC) [37] program to develop and deploy advanced measurement services to measure how the science and engineering community was taking advantage of NSF-funded research networks and exchange points. The original deployment included working with, and gathering data from, the seven funded IRNC projects: Atlantic Wave Software Defined Exchange [32], America’s Lightpath Express and Protect (AmLight ExP) [33], Networks for European, American, and African Research (NEAAR) [82], Pacific Islands Research and Education Networks (PIREN) [39], Pacific Wave [24], StarLight [43], and TransPAC4 [80], in addition to a set of science data archives. The international projects share the NetSage International Portal Bandwidth Dashboard [53], as shown in Fig. 2. Other individual resource sets, such as the Great Plains Network (GPN), iLight (the Indiana state network), KINBER, and so on, have a Portal specific to their data. In addition, the NetSage *All Data Portal* [57] includes the complete set of data collected for all of the other Portals, which is useful to analyze traffic more broadly, for example, by a science project that spans many networks globally. Table 1 lists the different data sources collected for each deployment. Note that perfSONAR test points are only available for the international resources and are not listed separately.

The data sources for 2020 included over 2.5 Billion flow data records collected from almost 60 sources, as well as SNMP data from over 60 unique nodes across the US and internationally. During 2020, over 3,600 unique users in 101 countries visited NetSage Dashboards.

**Table 1:** The list of the US domestic and international deployments for NetSage as of March 2020 and the number and type data sources collected from each for use in NetSage deployments

Project/resource set	SNMP	Flow	Science Archive
GPN	2	2	–
iLight	–	5	–
KINBER	–	2	–
FRGP/NCAR	–	1	1
SoX	–	3	–
TACC	–	1	4
PIREN, UH Astro	5	2	1
NERSC	–	–	10
ANA	7	–	–
Pac Wave/ CENIC	17	7	–
NEAAR/ NEA3R	3	3	–
TP4/ TP5	2	3	–
AmPath/ AmLight	11	6	–
StarLight	2	–	–

## Design methodology

The NetSage team adapted the Immersive Empathic Design Methodology (IEDM) [11] for developing visualizations. Visualization experts have commonly used this process, or similar techniques such as Design Thinking [9], to successfully produce effective visualizations for many decades. This methodology has eight steps:

1. Create profiles for representative stakeholders to understand their visualization needs.
2. Sketch storyboards to characterize the type of visualization to answer the stakeholder identified needs.
3. Present storyboards to stakeholders for feedback, which is often accomplished by recording storyboard presentations for stakeholders to view and comment on.
4. Update the storyboards based on the feedback from Step 3, and reiterate, as time and resources allow.
5. Develop prototypes based on the storyboards.
6. Give early working prototypes to stakeholders for them to try out in their own workflows.
7. Elicit feedback from the stakeholders.
8. Iterative development using the feedback to produce a successively better system, as well as to introduce additional requested features.

For example, for the initial NSF International Networks NetSage deployment, the profiles for representative stakeholders were defined by identifying the set of end users for the NetSage Dashboards and the types of questions they might ask of the data. The initial Dashboard users included:

- Network resource owners and operators who wanted to know the status of the resources;
- Collaborative research teams trying to understand resource use and how their data transfers would behave;
- Engineering staff to ensure effective resource use; and
- Staff members at funding agencies who needed additional insight into their investments.

After discussions with representatives from each audience, sets of use case questions were identified, including:

- What is the present state of the NSF-funded international network resources?
- What are the top sources or destinations for data flows using the NSF-funded international network resources?
- What are the top science domains that use the NSF-funded international network resources?
- What is the maximum, minimum, and average duration of large data transfers?
- Which countries are sharing data using the NSF-funded international network resources?
- Are there patterns of behaviors that can be identified about how the NSF-funded international network resources are used?
- Which sources or destinations have transfers that are not effectively using the NSF-funded international network resources?

These questions were used by the NetSage development team to design Dashboards with visualizations to provide the answers. A series of hand-drawn graphical storyboards were produced to describe the proposed Dashboards. The feedback during Step 3 enabled the NetSage development team to identify commonalities across the stakeholders and to adapt the Dashboard designs accordingly. This approach not only verified that user goals were being addressed, but also that each Dashboard was focused on addressing the response to a particular question.

As the use of the NetSage Framework expanded beyond the NSF-funded international network resources, we continued to deploy this design methodology and to incorporate feedback accordingly, leading to additional features for all end user stakeholders.

## NetSage architecture

The NetSage Software consists of a set of open source tools that follows a basic monitoring tool architecture, as shown in Fig. 2. *NetSage TestPoints* are a collection of software and hardware components that gather active and passive data into records that are sent to the *Data Ingest Pipeline*. The five-stage Pipeline filters those records and adds additional tags before de-identifying the data. The records are then stored in the *NetSage Archive*, a centralized storage framework consisting of two different databases, a Time Series Data System (TSDS) archive [89] and an Elasticsearch archive [20]. *Performance Dashboards*, built using the open source Grafana [31] analysis and visualization engine, access the records from the NetSage Archive and present visualizations to answer the questions identified by the stakeholders.

### NetSage data collection TestPoint

The core of the data collection process for a NetSage deployment is the set of hardware and software that make up the logical NetSage TestPoint. TestPoints use both active and passive measurement techniques to gather data for a broader understanding of network behavior.

TestPoints passively collect data from routers or switches using the Simple Network Management Protocol (SNMP) [10], an application–layer protocol for information about managed devices on IP networks, generally with a polling rate of once every 60 seconds. This data set includes the interface name, the number of input and output bits, any errors or discards, and data about unicast or multicast use.

The second type of passive data collected by the TestPoint is flow data from routers using tools such as NetFlow [51], sFlow [78], or IPFIX [12]. Flow data is typically sampled by a router at between 1:100 and 1:1000 packets. This data set includes information for sampled flows including the source and destination, the number of bits and packets transferred, the duration of the flow, the flow type, and the protocol and port used.

The third type of passive data collected by the TestPoint comes from packet header inspection tools running on science data archives using Tstat [45, 90], which was developed as part of the European Union (EU) Measurement Plane (mplane) FP7 project [88]. Tstat examines the packet headers for data flowing in and out of instrumented science archives and reports TCP statistics for each flow, including the congestion window size, the number of packets re-transmitted, the source and destination of the flow, the number of bits and packets transferred, the duration of the flow, the flow type, and the protocol and port used. Unlike similar data collected using a standard router flow tool, Tstat data is not sampled.

The fourth data set collected by the TestPoint is from active measurements using perfSONAR [76, 87], an open source network measurement suite designed to provide end-to-end performance metrics. There are currently over 2000 publicly registered perfSONAR nodes deployed worldwide [77]. NetSage deployments may include perfSONAR data sets for active measurements of throughput, latency, and loss.

Each of these data sets can be used in multiple ways. In the examples and figures that follow, we highlight which data sets are the source of the information given.

### Data Ingest Pipeline

Records from the NetSage TestPoints are sent to the Data Ingest Pipeline, which consists of five stages, as shown in Fig. 2.

In the first stage, records for flows smaller than a threshold are discarded. The threshold is generally set to the size of 10 MBytes over 5 min, but can be adapted on a per-installation basis. The primary goal of the NetSage Framework is to understand large-scale data transfers, so records related to small flows do not need to be retained. With a threshold at 10 MBytes, the number of records collected is reduced by 90$\%$, however we retain data for approximately 90$\%$ of the volume of the data transferred. This filtering has several additional benefits. It reduces both the CPU needed to run the Pipeline software as well as the overall storage space required by the NetSage Archive. It also increases the level of privacy, as flows related to emails or web page downloads are smaller than the threshold and therefore discarded.

In the second stage of the pipeline, we ensure that each record represents all of the data for a single flow by stitching together the existing multiple records for longer flows. Most flow collection techniques share data at specified time intervals, generally 5 min. If a single data transfer occurs over several time intervals, multiple partial records will be created, one for each time frame. We stitch these together to ensure each record represents a full transfer.

At the third stage, tags are added to the record to map the source and destination of the data transfer to their Autonomous System (AS) Numbers and Names [4]. Previously, the MaxMind GeoIP database [44] was used, but in December 2020, the NetSage Data Ingest Pipeline shifted to using the Center for Applied Internet Data Analysis (CAIDA) AS to Organization Mapping Data set [8], which maintains better human-readable naming. To identify institutions that do not have their own ASN, the Pipeline uses information from the Shared Whois Project (SWIP) [84]. The SWIP data is used to formally document cases where subsets of IP space need to be identified. For example, a regional network may allocate a piece of its own IP space to a member institution. SWIP enables the traffic to that IP-subset to be properly attributed to the institution and not the regional network, which in turn allows the NetSage Dashboards to list the sources and destinations more accurately.

In the fourth stage, tags are added to the record to identify the science domain and project information using the NetSage Science Registry [55]. One of the primary use cases for NetSage is to better understand which science domains and projects are using a set of networked resources. In order to do this, we need a mapping of IP address spaces to specific science projects and domains. We created the Science Registry to do this mapping. The system supports collaborative and crowd-sourced data entry. With this system, we can add additional tags to each Flow record as needed, including the research project name, the science domain, the universities or institutions involved with the project, geo-location data, or other related data. As of April 2021, the NetSage Science Registry contains over 430 entries for over 370 Organizations and over 250 Science Projects. The Registry is continually updated by the NetSage team and its collaborators.

In the fifth and final stage, the low order bits of the IP addresses are stripped off to de-identify the data. One of the system design goals of the NetSage Framework was to avoid storing personally identifiable information (PII), and this stage of the pipeline addresses that requirement. For IPv4, the 8 low-order bits are removed. For IPv6, the 64 low-order bits are removed. Full details are given in the NetSage Data Privacy Policy [54], which was developed to balance the need for user privacy with the practical value of the data. Note that since the NetSage Archive does not include full IP addresses, there is no reference data related to a person or at a personal level, so this approach is compliant with the European Union General Data Protection Regulation (GDPR) [25] as well.

Currently, the Data Ingest Pipeline can be run in two ways—at a collection point at Indiana University or in a container on a system owned by the resource owner. The data is simply pointed to the correct collector. By using the containerized deployment, a resource owner can further control the data sharing process. Current NetSage deployments are split roughly 50–50 in how they deploy the Data Ingest Pipeline.

### Security of NetSage Ingest Pipeline

As with any network monitoring system, the overall entire system must follow security best practices to ensure that data is not tampered with and to ensure that only the intended data is ever published. The NetSage deployment at Indiana University follows the Defense in Depth [18] philosophy. All components require two-factor authentication to access. All data transfers are via SSH connections. When the NetSage team works with other sites to access monitoring data, a security review is undertaken.

Also, as mentioned above, only public data sets that are not considered sensitive are published in public Dashboards. In cases where there are privacy concerns for the data, the NetSage Dashboards are secured by password or Shibboleth.

### NetSage Archive

After passing through the Data Ingest Pipeline, the data sets are stored in the centralized NetSage Archive. The Archive consists of a Time Series Data System (TSDS) archive and an Elasticsearch archive. The Indiana University OmniSOC [75] hosts the NetSage Archive and provides production-quality security and support for the data resource.

TSDS was developed by the Indiana University GlobalNOC [27] to provide an efficient way to store data with consistent time intervals. It provides well-structured and high-performance storage and retrieval of time series data and metadata, in our case, SNMP and perfSONAR data.

The Elasticsearch, Logstash, and Kibana (ELK) Stack is open source software that forms a scalable system used to flexibly ingest, store, and analyze sporadic event data. The Elasticsearch archive stores data as JSON documents and indexes it for quick searching and retrieval. The NetSage Archive uses the Elasticsearch archive to store flow data and data from Tstat. One of the features of Elasticsearch is that it is designed to be horizontally scalable, meaning that both the performance and capacity of the Archive can be increased by adding more nodes to the cluster running the NetSage Archive.

The NetSage archive is structured so that if other databases of information were needed, they could also be included in the archive. In this way, each type of data can be stored so that access to the data itself is done efficiently.

### Dashboard Visualization Components

NetSage Dashboards are used to visualize the answers to the stakeholder questions that were identified as part of the design methodology. A set of NetSage Dashboards for a particular suite of resources is referred to as a *NetSage Portal* for those resources. The Dashboards are built using the open source Grafana analysis and visualization engine. Each Dashboard consists of a set of *Visualization Components* that show different aspects of the data in response to a query. In cases where Grafana did not have a ready-made Visualization Component, new ones were developed using D3.js [15], and contributed back to Grafana when possible. In some cases, the following Figures include only partial Dashboards. Each caption includes a reference to the exact URL to see the full Dashboard if needed.

The NetSage Visualization Components range from simple to complex, each included as part of a given Dashboard to tell its part of the story to meet a design goal. On the simple end of the scale, basic line and bar charts are used to answer stakeholder questions such as “what is the present state of a resource?” in several Dashboards, such as shown in Fig. 2.

**Fig. 3:**
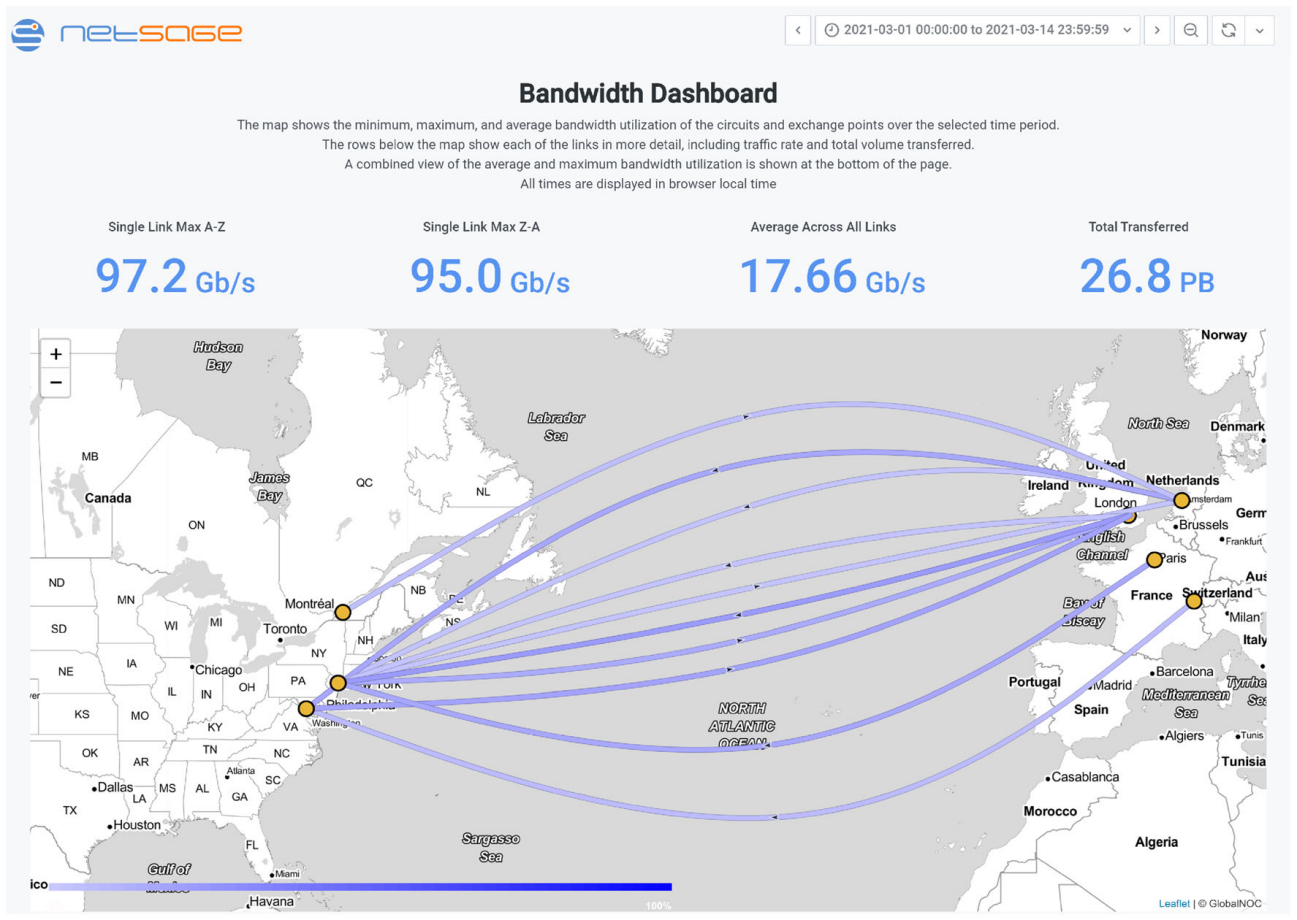
The ANA Portal Bandwidth Dashboard [59] shows SNMP data for the ten circuits that are part of the ANA consortium using a map that allows easy inspection

We use *maps* to show geographical relationships for different data sets. For example, the Advanced North Atlantic (ANA) Network [1] uses the ANA Portal Bandwidth Dashboard in Fig. 3 to enable stakeholders to easily visualize and compare the volume of traffic between physical end points. In this case, it can also be used to easily see if load balancing across the links is taking place based on the collected SNMP data.

**Fig. 4:**
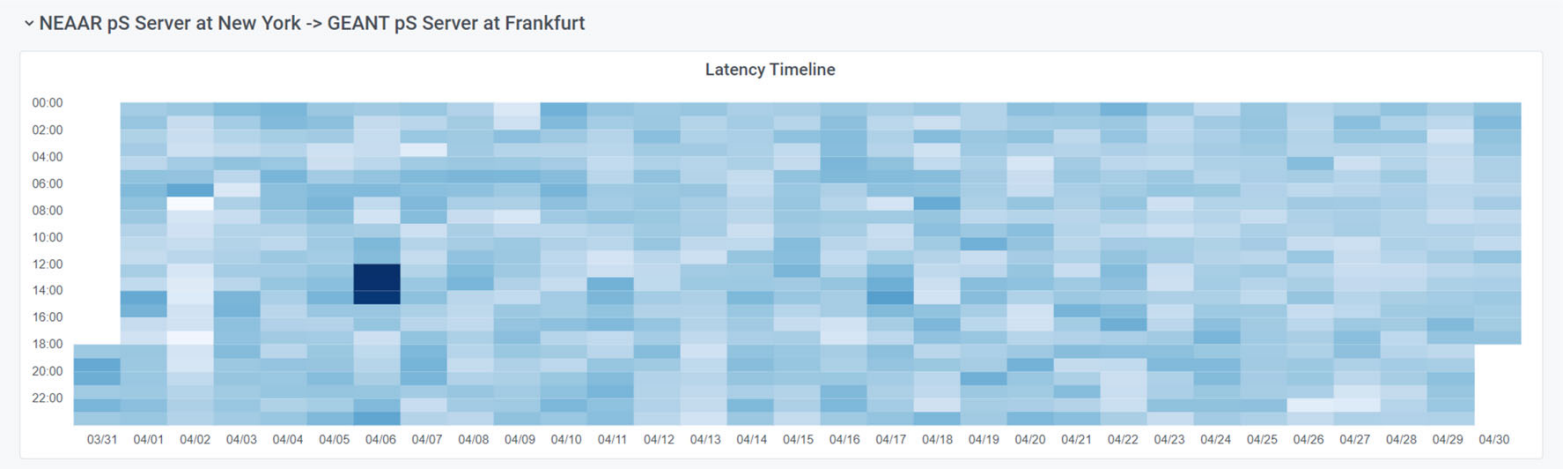
A Heatmap showing perfSONAR latency data, which is part of the International Portal Latency Pattern Dashboard for tests between the ManLan and GEANT Open London exchange points over the NEAAR link [65]. The x-axis is the day of the month and the y-axis is the time of day. Darker colors indicate larger amounts of latency

We use *Heatmaps* to show changes in values over time, where the x-axis is time and a darker color indicates a larger value. Heatmaps can answer stakeholder questions such as “Are there patterns of behaviors for the use of the resources?”. This type of display can accentuate changes of behavior over time, as seen in Fig. 4, which shows the International Portal Latency Pattern Dashboard for the NSF-funded NEAAR link using perfSONAR active measurement latency data.

**Fig. 5:**
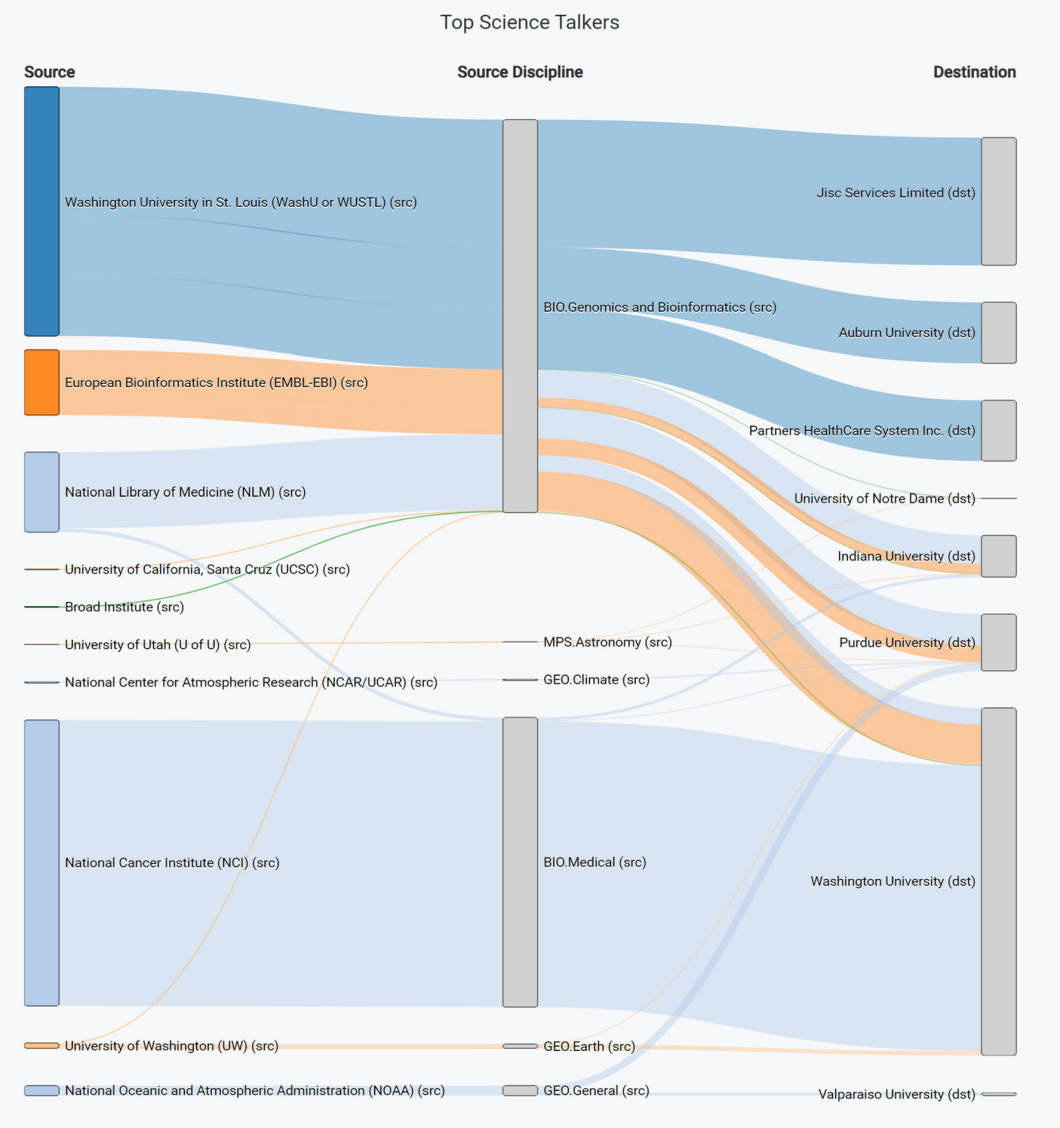
A Sankey graph showing which institutions are sharing data for identified science domains on the iLight network [63]. The width of the ribbon is proportionate to the volume of flow data sent

*Sankey Graphs* [79] are used to show relationships between items using a ribbon graphic, where the width of the ribbon indicates the quantity proportionately. We use Sankey Graphs as a visual way of answering stakeholder questions such as “Which institutions are sharing different types of science data between them?”, as shown in Fig. 5 for a set of science domains using the iLight Portal Science Discipline Dashboard.

**Fig. 6:**
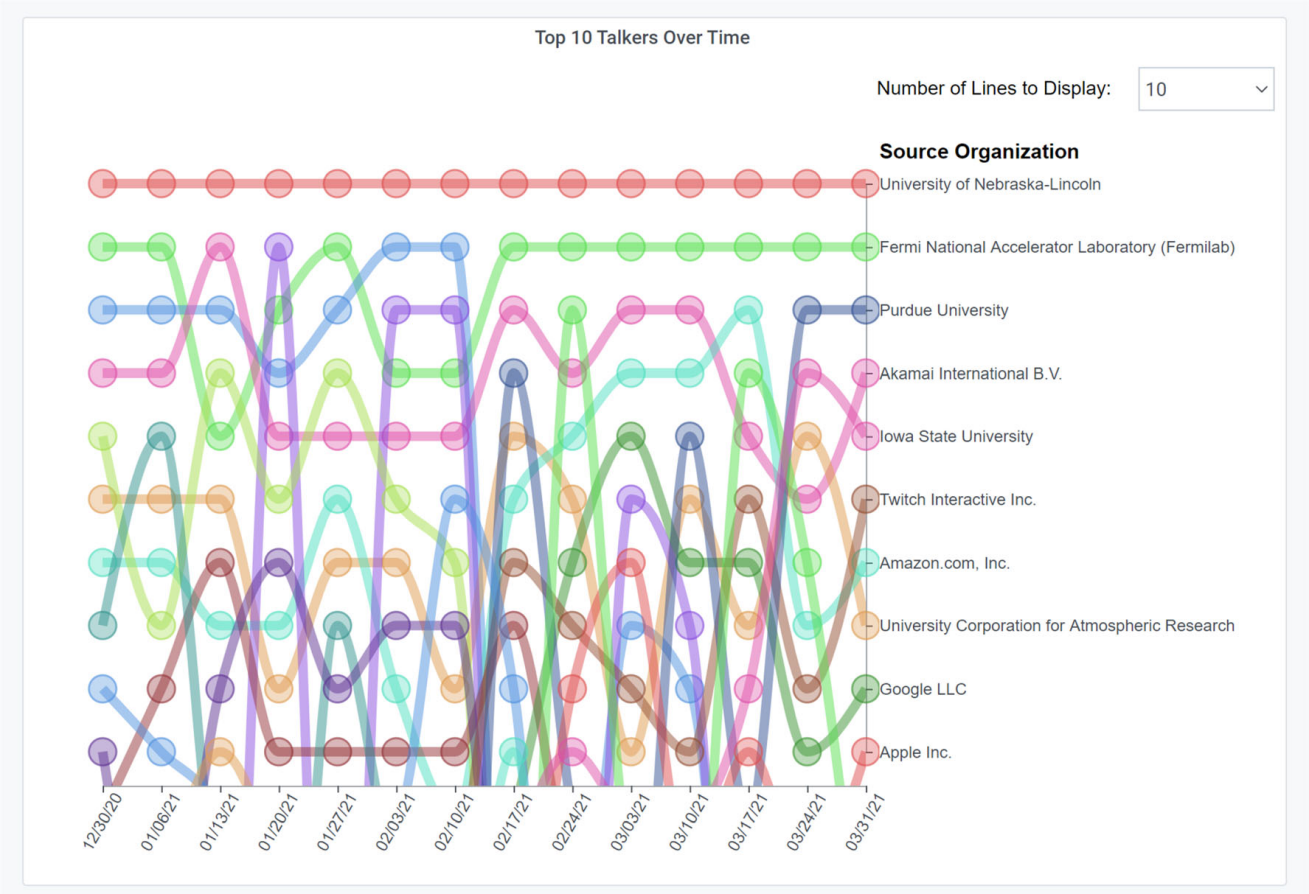
A Bump chart showing the GPN Portal over three months [62]

A *Bump Chart* [92] is a special form of a line plot that is well-suited for exploring changes in rank over time. We use Bump Charts to compare the rankings of Top Talkers over time, as shown in Fig. 6. This allows end users to easily see multiple observations with respect to each other, rather than the actual values itself. For example, the figure shows that for the Great Plains Network (GPN), energy science data transfers for the CMS experiments, with data sources at UNL and FermiLab, dominate the list consistently.

**Fig. 7:**
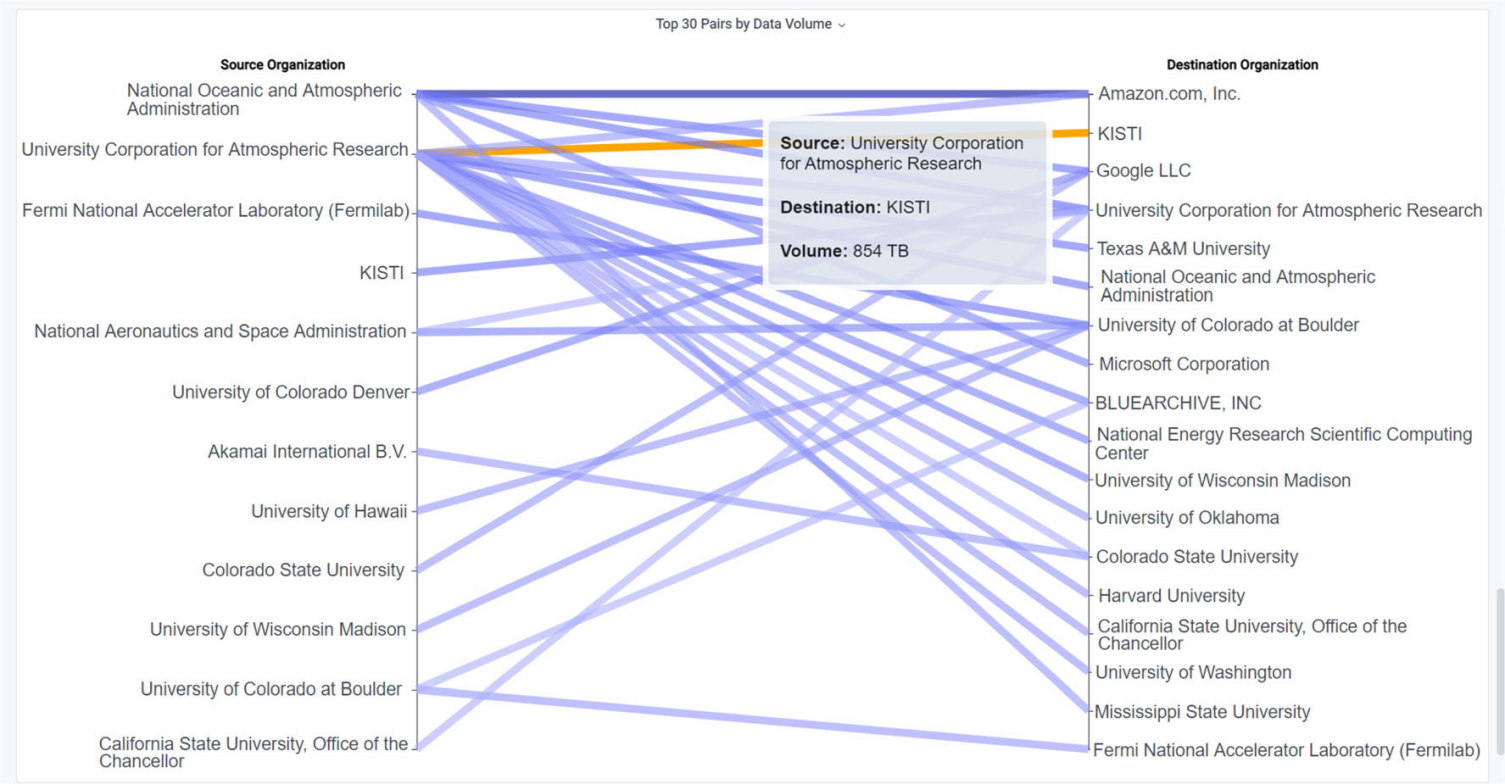
A Slope Graph from the FRGP Portal Flow Data Dashboard showing the relationship between sources and destinations [60]

Finally, a *Slope Graph* [91] allows the direct visualization of the relationship between two variables. For NetSage Dashboards, Slope Graphs are used to show relationships between Sources and Destinations of flows. For example, Fig. 7 shows the Slope Graph for the FRGP Portal Flow Data Dashboard and visualizes the relationships between sources and destinations. While the bar charts (also included in the Dashboard) indicate that NOAA and NCAR/UCAR share more data, their pervasiveness as sources is more clearly described with the Slope Graph Visualization Component.

### Other uses of the NetSage framework

#### ESnet peering decisions

The NetSage System is flexible enough to be applied in several other use cases. At the Energy Sciences Network (ESnet) [23], there was a need to replace a legacy flow collection system to serve the network engineers in tasks such as general capacity planning, peering analysis, and determining when to establish a direct connection to a network and at what capacity. In order to meet this need, it was decided to augment the NetSage Data Ingest Pipeline and NetSage Archive to also include information from the Border Gateway Protocol (BGP) [6]. This data set includes information about all the networks involved in delivering packets in a flow to their destination once they leave the local network. This is significant because it includes not just the source and destination networks, but intermediate networks as well.

The ESnet development team is using the standard NetSage codebase in GitHub [52] for their enhancements, which will enable each deployment to leverage the common components for their separate use cases.

#### DDoS detection on international backbones

The International Networks at Indiana University (IN@IU) [35] team has been using components from NetSage with slight alterations to detect Distributed Denial of Service (DDoS) attacks. This type of attack generally consists of an attacker compromising a number of hosts that are directed to send large amounts of data to a target system in an effort to overwhelm the target. Many types of DDOS attacks have traffic patterns that are easy to identify through the examination of packet header samples via flow data.

Unlike the current Data Ingest Pipeline that removes data about all flows under 10MB, this use case requires all data and also full IP addresses for the flows. Because of this, the Dashboards must also be locked down so that the data is kept secure. The NetSage team worked with members of IN@IU to adapt these components, and a new Dashboard was developed as well. The Dashboard displays the top sources, destinations, and flow pairs by highest number of flows under a defined size to identify the possibly suspect flows. It also includes a panel that shows the top single destination of these small flows along with all of the associated sources, aiding in quick identification of a potential DDoS target.

## Design lessons learned

In the course of developing any large-scale pragmatic software framework, plans change and lessons are learned. Three of the major lessons as we have experienced while developing and deploying the NetSage Framework have been to adapt when necessary, to leverage other people’s work as much as possible, and that what users request will change as soon as they have a prototype to work with (sometimes referred to as “No plan survives contact with the enemy”—Helmuth von Moltke).

### Lesson 1: Adapt when necessary

Sometimes, a change of plans can result in an improved approach. The NetSage development team had originally planned to collect both sampled and unsampled flow data from routers (not science data archives) by using a packet-header inspection software tool such as ARGUS [3], Zeek (formerly called Bro) [94], tcptrace [86], or Tstat. This approach would have enabled a comparison between the different measurement approaches. However, part of how these types of packet-header inspection tools function is that they track both sides of the “conversation” between a source and a destination for each data transfer. In order to track both sides of the conversation, data sent from the source to the destination must use the same path through the network as when data is sent from the destination to the source. It was discovered that many, if not most, international data transfers experience asymmetric routing. In other words, the network path from the source to the destination was not the same as from the destination to the source. Because of this, none of this class of packet-header inspection tools could be used in the middle of the path at a router.

However, packet-header inspection tools would be able to gather data when they were placed at the end of a path, that is, at the actual source or destination. We evaluated the common sources and destination of data sets in the US and identified several major data centers. We then worked with them to deploy Tstat and a TestPoint to be able to pull the data into the NetSage Archive. We are now collecting data about transfers to and from science archives at the National Energy Research Scientific Computing Center (NERSC) [50], the Texas Advanced Computing Center (TAAC) [85], the National Center for Atmospheric Research (NCAR) [49], and the University of Hawaii’s Institute for Astronomy (IFA) [34].

### Lesson 2: Leverage when possible

The NetSage development team always planned to leverage other open source projects as strongly as possible in order to maximize project resources, in part also looking toward other NSF-funded projects it could utilize. For example, the initial NetSage Archive implementation used the existing TSDS database. When the data collection expanded to include flow data, the NetSage Archive was updated to include the Elasticsearch archive as well, as opposed to building a tailor-made one in house. Our initial implementation of the Data Ingest Pipeline used NF Dump [73] and custom scripts, which over time have been transitioned to taking advantage of logstash [40] instead. We also shifted from the commercial MaxMind database we were using to map IP addresses to organizations to use the NSF-funded CAIDA AS to Organization Mapping Dataset. Similarly, the initial NetSage NSF-funded international network Portal Dashboards and Visualization Components were written using custom software, because when the project started there was no clear best toolkit approach to building them. In Year 3, we shifted to using Grafana, which has saved countless hours of development and decreased our support burden.

### Lesson 3: Changing requests

The NetSage development team, like most builders of pragmatic software, has also discovered there are successes and opportunities when working directly with an active user base. For each new Dashboard that has been story-boarded, designed, and deployed, once the stated requirements were met, the stakeholders will often take the opportunity to request additional functionality. An ongoing challenge has been to keep the Dashboards focused and simple enough for use by a wide audience, while adding the requested additional data visualizations. We continue to expand the use cases we address and the visualizations used to meet stakeholder needs.

## Discoveries made using the NetSage framework

The NetSage Framework is used daily to interpret a variety of networking and data transfer behaviors by resource owners, science teams, and network engineers to better understand the performance and patterns involved in a wide area data movement. End users can work with NetSage Dashboards to gain insight into the data movement at the system, institution, or project level, and to see longitudinal changes in behavior in a broad set of situations.

### How a system of resources is used

**Fig. 8:**
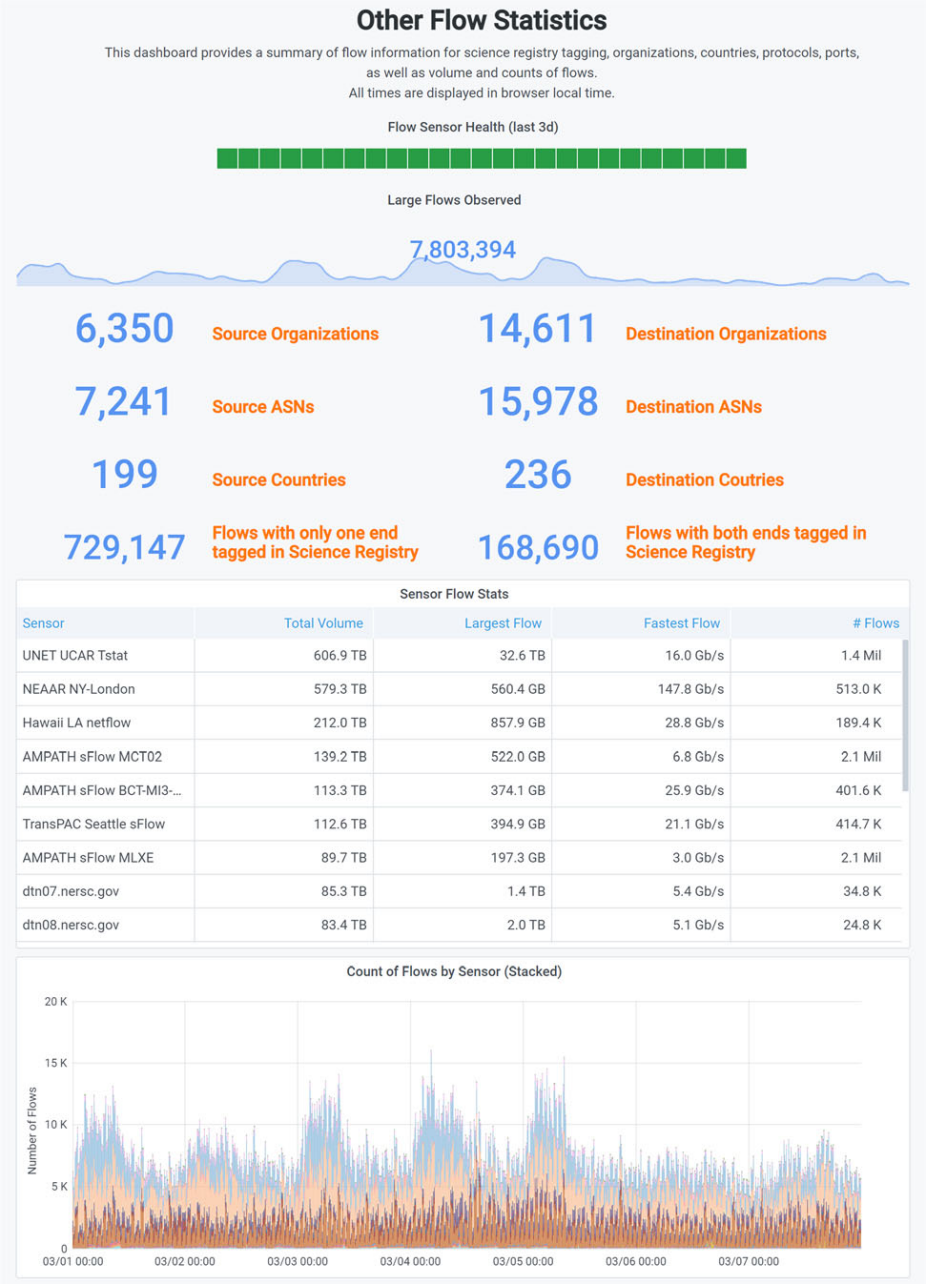
A portion of the International Portal Flow Statistics Dashboard for the first week of March 2021 [67] showing the overall statistics for the use of the NSF-funded international networking resources

The use case that the original International Portal was developed to address was to better understand how NSF’s multi-million dollar investment in international networks was being used by the US R&E community. The NetSage team initially developed two, high-level Dashboards to give basic details about the use of a resource, in visual graphs and in summary statistics. The Bandwidth Dashboard, shown in Fig. 2 for the International Portal, uses SNMP data to generate and display a map of the resources, details about the use of each circuit, and summary line graphs for the average and maximum bandwidth utilization. In addition, the Summary Statistics Dashboard, shown in Fig. 8, highlights basic numerical data about the resource using SNMP, Flow, Tstat, and Science Registry data. It gives a high level overview of all the resources in a Portal in this way.

**Fig. 9:**
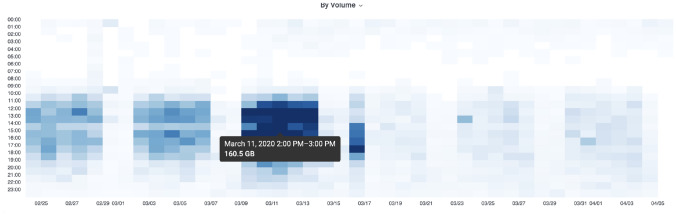
A Heatmap from the Pacific Wave Portal Flow Data Dashboard that displays data related to transfers with one end point at the Zoom video conferencing facility that crossed the Pacific Wave Exchange Point to an academic institution source or destination during February–April 2020 [69]. The x-axis is the day of the month and the y-axis is the time of day

However, NetSage Dashboards can also take advantage of other components to answer several different questions about usage. For example, Fig. 9 shows a Heatmap from the Pacific Wave Portal Flow Data Dashboard that uses flow data to measure data transfers to and from the Zoom video conferencing hosting site during the time frame where R&E institutional use of Zoom changed radically. In March 2020, when universities started to responding to the COVID-19 pandemic-related restrictions, network resource owners needed to know how their systems were responding to the change of use. The Heatmap shows data volumes starting in February that increase on/around March 12 when many US universities declared that researchers could not travel. This was followed 10 days later by a decrease, likely caused by a combination of institutions shifting to Spring Break, institutions issuing work from home directives (so the traffic shifted to home networks not R&E networks), and Zoom shifting some of its hosting to use cloud services, rather than their own IP space.

**Fig. 10:**
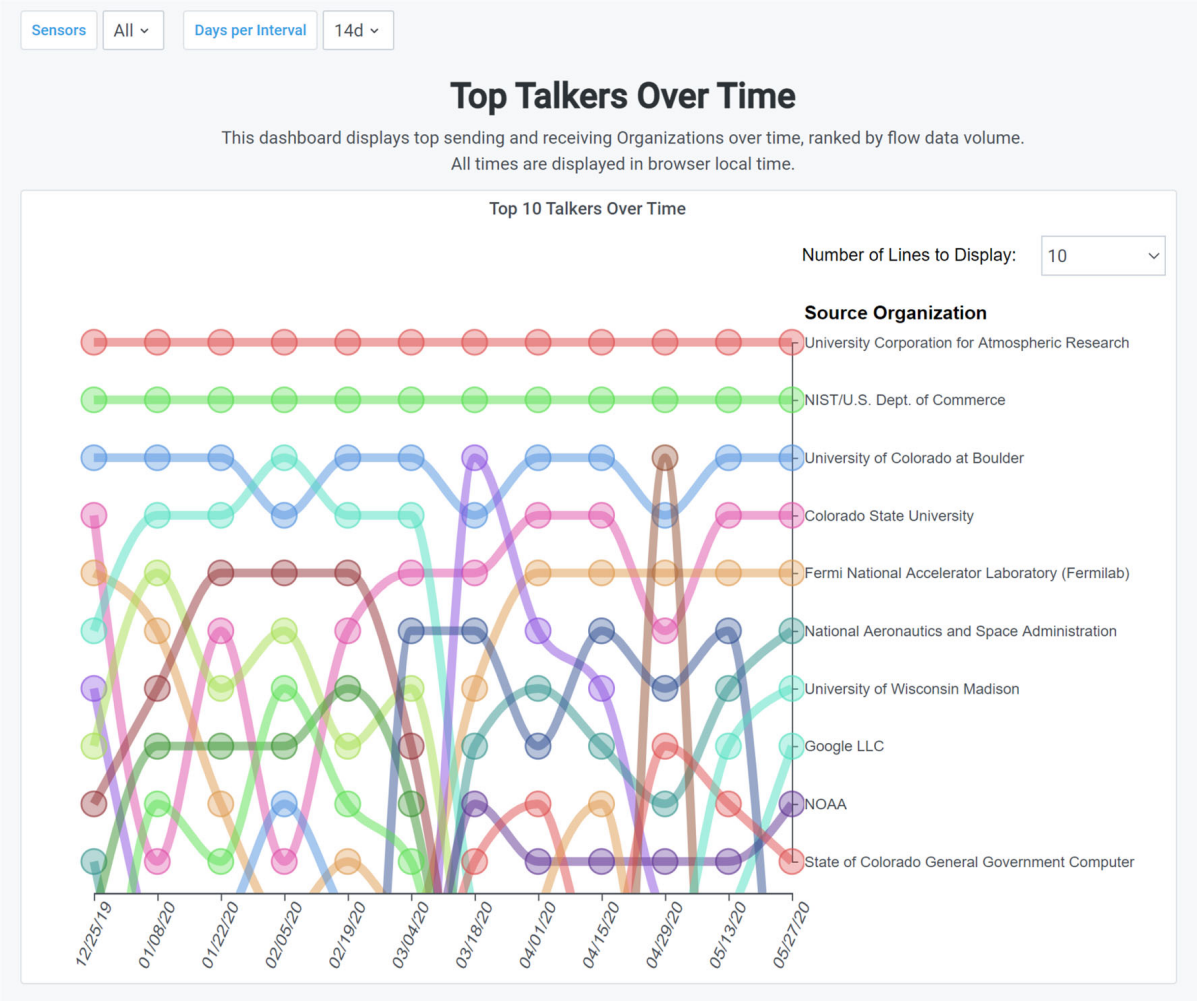
The FRGP Portal Top Talkers Dashboard for January through June, 2020 [61], showing how the Top Talkers on R&E networks changed radically during the shift in workspace when COVID-19 related restrictions were put in place in early March

Another example of changes at a system level that took place during the March 2020 time frame can be seen in Fig. 10. Its common for regional networks to want to track the use of their network over time, which is why NetSage has a Top Talkers over Time Dashboard. In this example, the FRGP Portal Top Talkers Dashboard for January through June, 2020, shows that while the three highest Top Talkers stay the same, a noticeable shift happens in early March, at the time that US educational institutions implemented pandemic restrictions. Note the large amount of change during March.

### How the resources of an individual institution can change

**Fig. 11:**
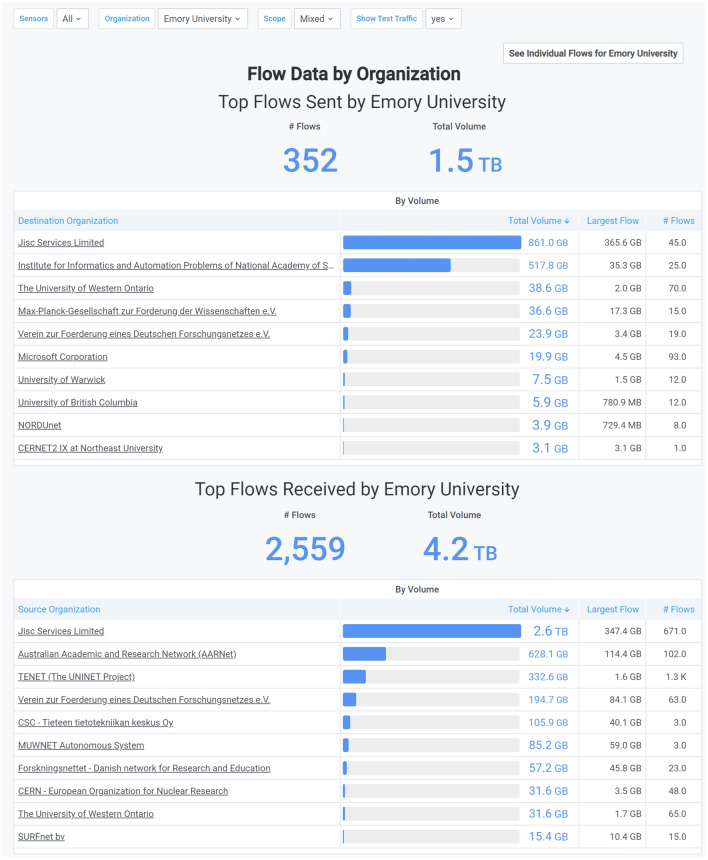
SoX Portal Flow Data by Institution Dashboard [70] showing which international institutions send or receive data from Emory University for January–March 2021

Often, an individual institution will want to examine the traffic it sends and receives to better interpret how the institution’s researchers are working with collaborators. We created the Flow Data by Institutions Dashboard to answer the core questions asked from that point of view. For example, Fig. 11 shows data for Emory University as part of the SoX Portal Flow Data by Institution Dashboard. Specifically, we selected the filters for this Dashboard to show how Emory interacted with international institutions over a 3 month period. During this time, for example, we can see that Emory receives almost three times more data than it sends, and that the researchers are working with a wide variety of international collaboration sites. However, for both sending and receiving data, the main collaborators are in the United Kingdom, as indicated by the listing for JISC, the national R&E network for the UK.

**Fig. 12:**
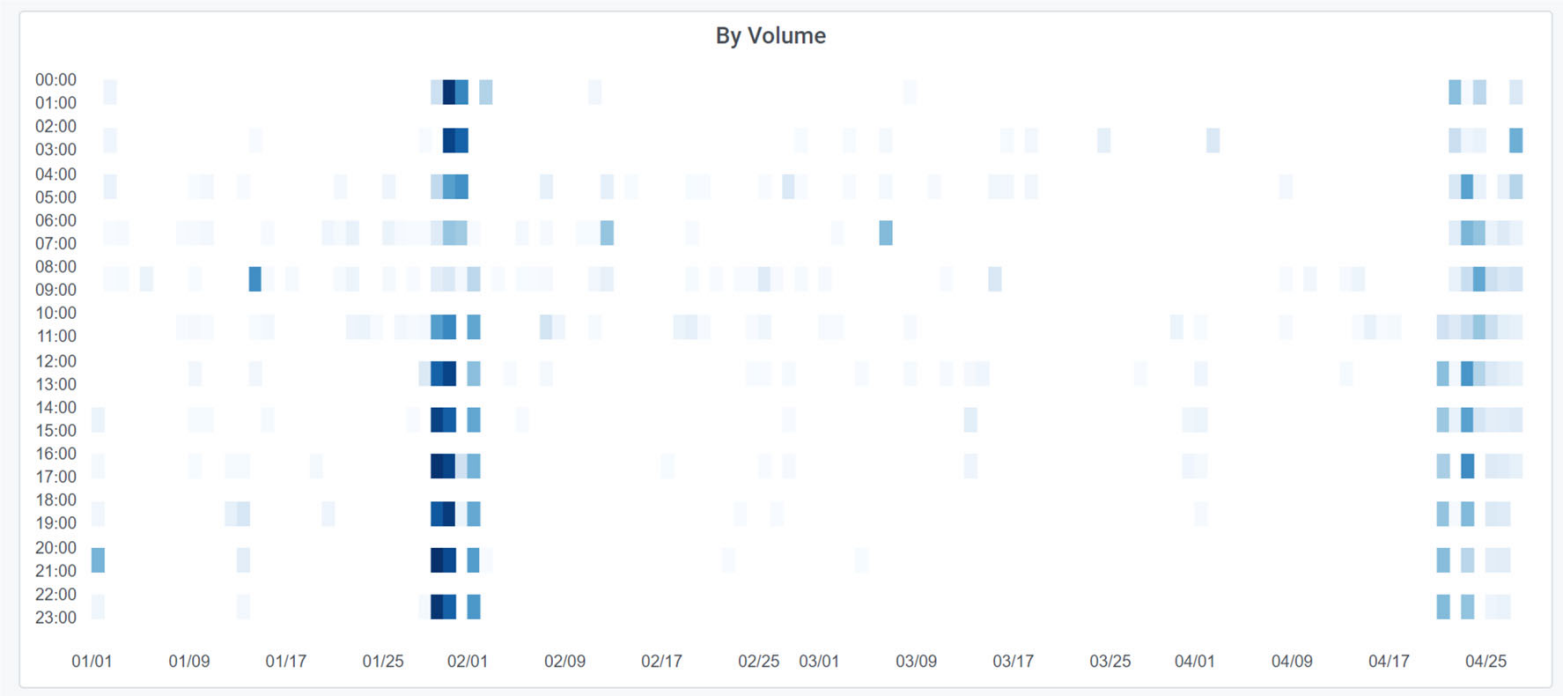
A Heatmap showing data transfers from the the National Library of Medicine in early 2020, as part of the KINBER Portal Individual Flows Dashboard. [68]

In another example, the National Library of Medicine (NLM) hosts many bioinformatics related data sets, of particular interest to pandemic research in 2020. Figure  12 shows a Heatmap of data transfers with one end at NLM using flow data as part of the KINBER Portal for Individual Flows for the first five months of 2020, and notable are the large transfers in January and late April.

### How the resource use of a project can change

**Fig. 13:**
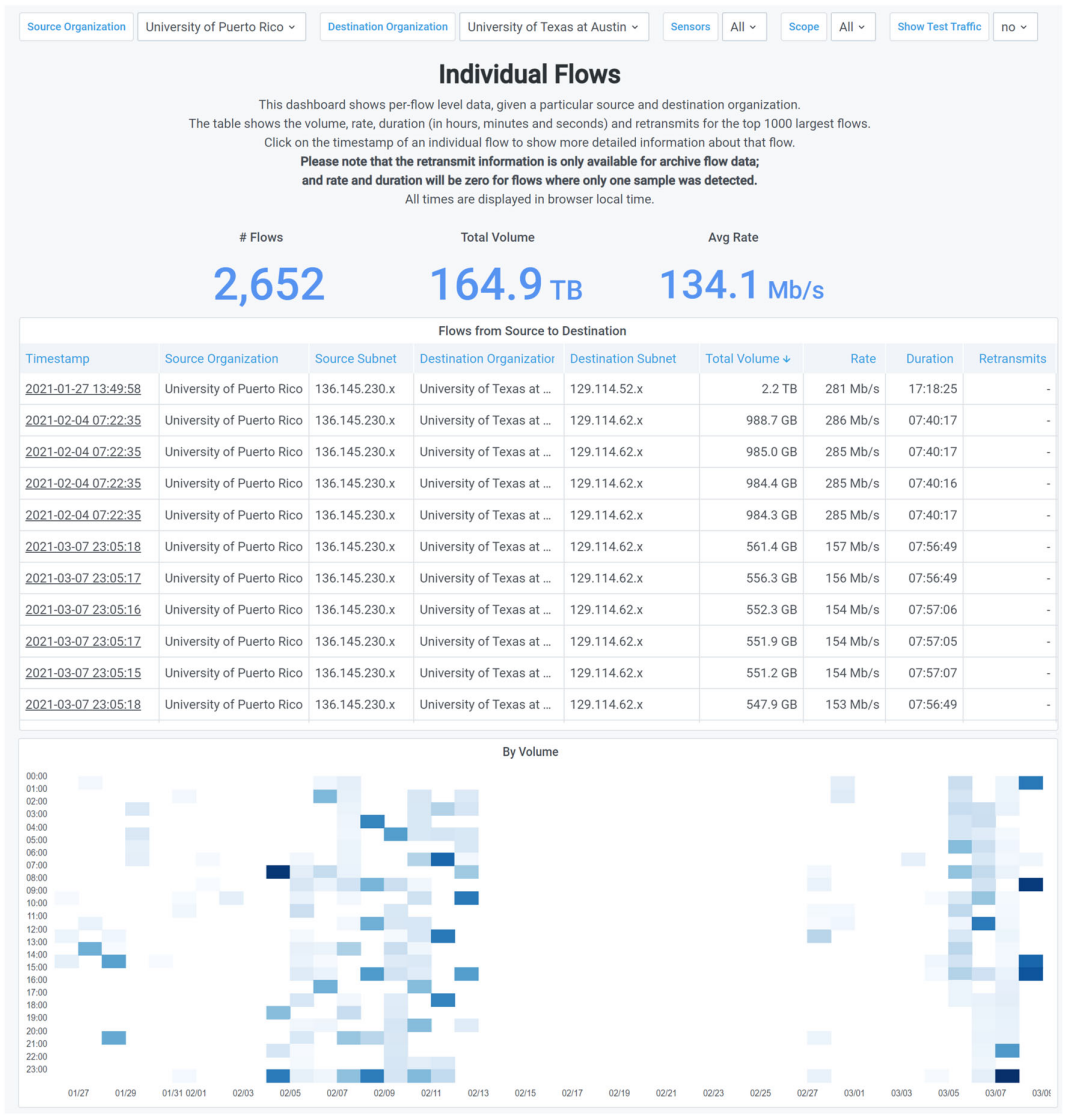
The TACC Portal Individual Flows Dashboard showing data transfers from the Arecibo Observatory in Puerto Rico to the TACC science data archive [71]

NetSage Dashboards can also be used by specific science projects to explore how their data transfers are performing and who is accessing their science data archives. For example, the Engagement and Performance Operations Center (EPOC) [22] often uses the US domestic NetSage deployments to evaluate behaviors of data transfers as part of its Roadside Assistance process [22]. The EPOC team was part of a joint collaboration with partners at TACC, University of Southern California, and Globus, to transfer massive astronomy data sets from the Arecibo Observatory [2] in Puerto Rico to the TACC science archives [14]. The TACC Portal Individual Flows Dashboard enabled engineers to visualize the progress and speed of the the transfers by visualizing the flows between the two institutions, as shown in Fig. 13.

**Fig. 14:**
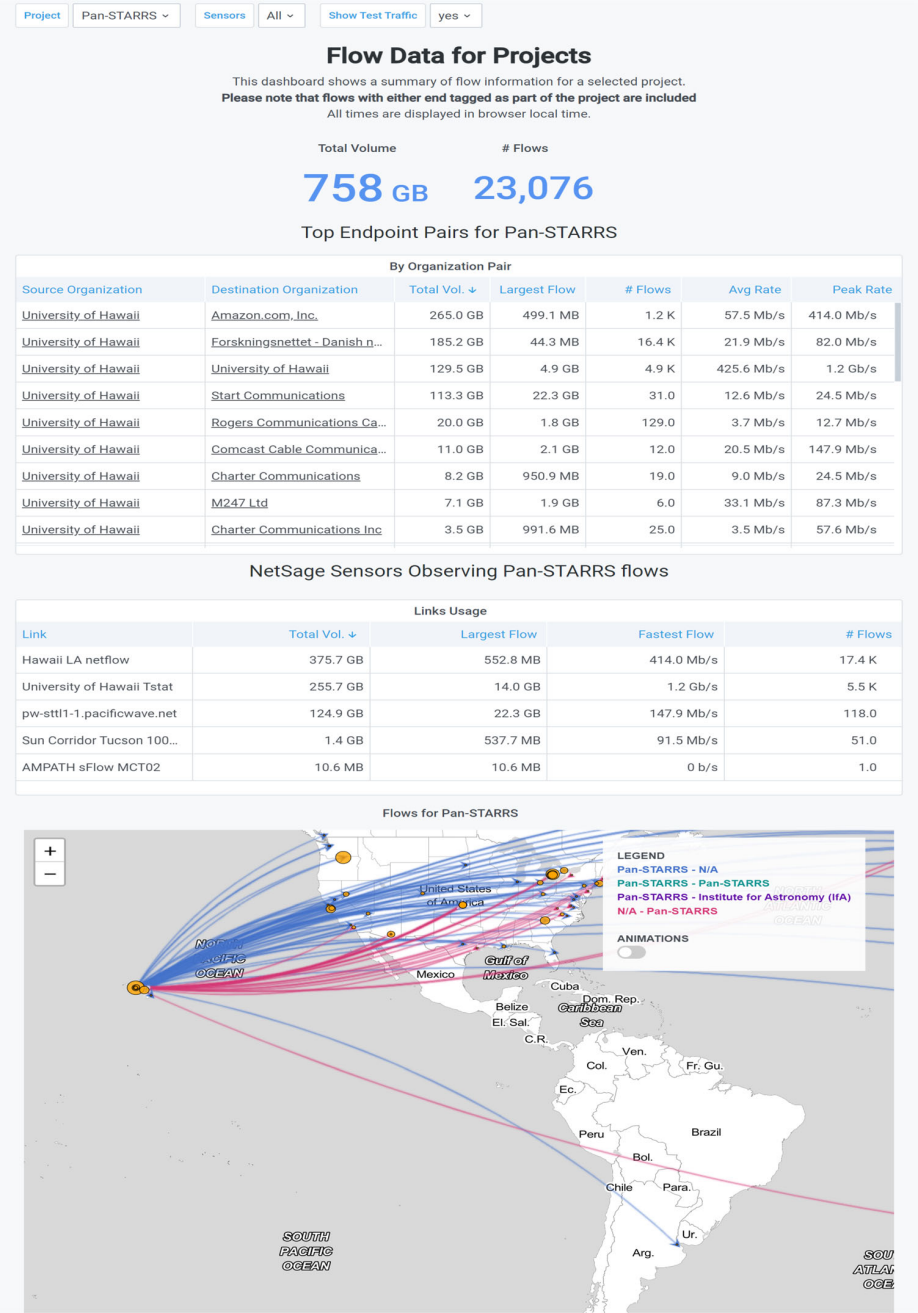
The All Data Portal Project Dashboard for PAN-STARRs during February 2021, showing how collaborators are accessing the data sites primarily in Hawaii [58]

The PAN-STARRS collaboration has used the All Data Portal Project Dashboard for PAN-STARRS to examine the associations between various R&E entities, some expected, others unexpected. The table of sensors in this Dashboard, shown in Fig.  14, indicates where the data for the Dashboard was collected and gives a sense of the network paths that are involved when sharing PAN-STARRS data. Scanning that table shows that there is relevant traffic on AMPATH, a collector in Miami that generally shows data traveling between the US and South America. A quick check of the map shows that the South American contact is part of Associación Redes de Interconexión Universitaria (ARIU), the national R&E network for Argentina, rather than one of several Chilean astronomy sites, as might be first assumed.

### Identifying changes or unexpected traffic

**Fig. 15:**
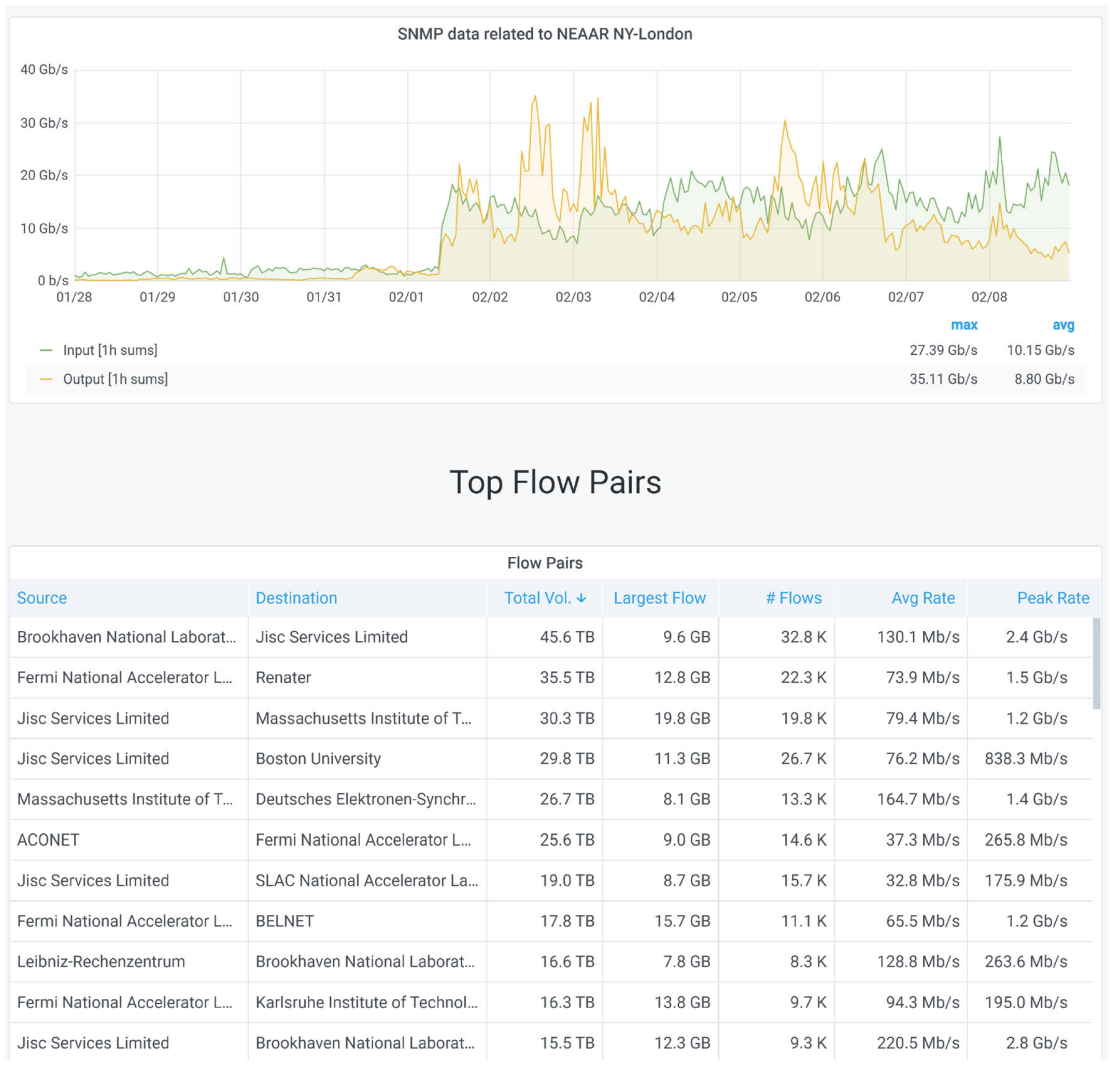
Traffic volume graph using SNMP data to show the increase in network traffic on the NEAAR link between New York and London for January–February 2018 [66]. Note the two colors indicate traffic in different directions on the circuit

Resource owners use their NetSage Portal to track typical behavior and to identify when behavior changes occur. When unexpected changes are observed, this may lead to further investigation. For example, Fig. 15 shows part of the Analysis Dashboard for the International Portal for the NEAAR project, the NSF-funded network collaboration between the US and Europe. End users can utilize an Analysis Dashboard, such as the one shown in Fig. 15, to relate SNMP data changes with the associated flow data to identify what transfers may be causing the shifts in behaviors. A significant change in behavior was experienced by this link in early 2018 because the US Department of Energy network operators added the NEAAR circuit to the set of network resources that support data transfers related to the Large Hadron Collider (LHC). In this particular case, NetSage Dashboards were able to exhibit this change even before the email notification was sent to the owner of the NEAAR circuit.

**Fig. 16:**
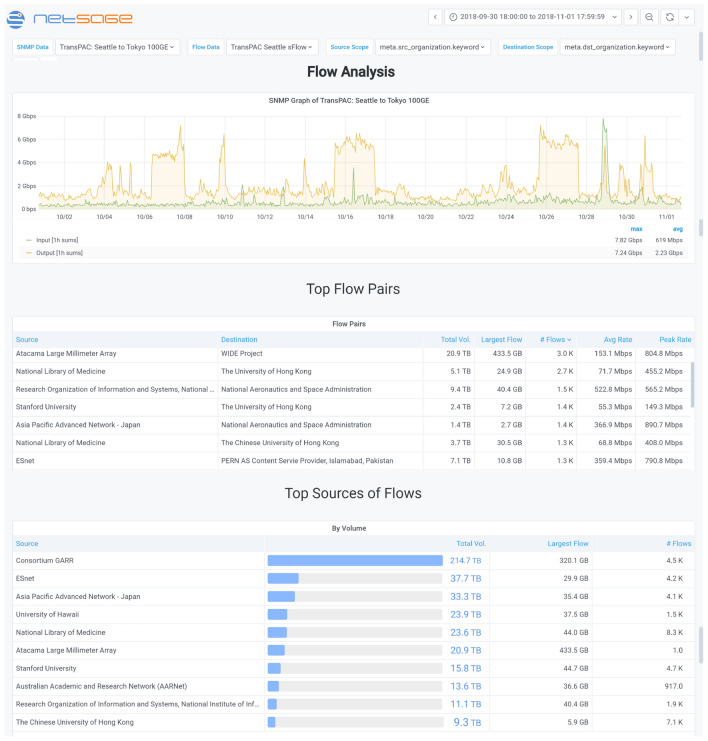
A partial flow analysis [72] Dashboard showing SNMP and flow data that identifies recurring data transfers that were part of a VLBI astronomy research project between Italy and Japan over the TransPAC4 link between Seattle and Tokyo in October 2018

Another example of unexpected traffic patterns was seen on TransPAC4. TransPAC4 is the NSF-funded international network project that supports connections between the US and Asia. Engineers for TransPAC4 used the International Portal to identify an unusual pattern of behavior where every 10-12 days there was a significant increase in the data volume over the network resource, as shown in the partial screen shot of the Analysis Dashboard in Fig. 16. Investigation indicated that the periodic data transfers were taking place between the Instituto di Radioastronomia, in Italy, and the Kashima Space Technology Center in Kashima, Japan, and that the traffic was related to an astronomy very-long-baseline interferometry (VLBI) project. The workflow for VLBI applications involves several geographically distributed radio telescopes that are all aligned on the same celestial object, in this case, all located in Italy, sending their data to a collector site, in this case in Japan. Identifying large scale data movement patterns such as this can also give resource owners an opportunity to work with end-user scientists to identify potential performance options. For example, in this case, additional investigation took place to ensure that this path was the most efficient for the collaboration since there were several different routing options.

### Identifying possible routing errors

**Fig. 17:**
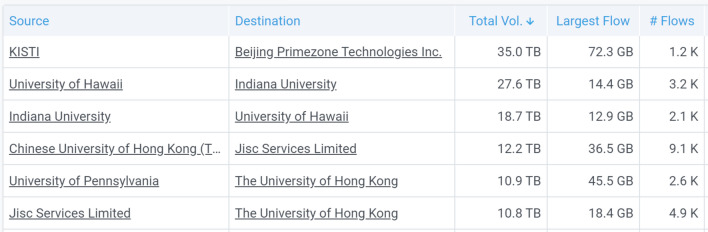
A partial listing, from collected flow data, of the top pairs of organizations transferring data over the TransPAC4 connectivity between Guam and Hong Kong for September-December 2018 [64]. This table shows that over 35 TB of data was incorrectly moving from KISTI in South Korea to China

NetSage is one of several tools that can also be used to identify when data transfers are not takeing the part or route that is most efficient. For example, when additional network capacity comes on line, it is common to see routes change, often in unexpected ways. One example of this was seen when the TransPAC4 project added a new 20G connection between Guam and Hong Kong. The partial Dashboard, shown in Fig. 17, indicates that the top pair of organizations transferring data over the new connection was the Korea Institute of Science and Technology Information (KISTI) in South Korea and the Chinese Academy of Science (identified as Beijing Primezone Technologies, Inc. due to its IP space). Traffic between these locations clearly should not be going over links to the USA. A deeper investigation showed the traffic was routed across the Pacific Ocean twice, resulting in significantly decreased performance. A discussion with the network engineers overseeing different parts of the path determined that the routing preferences were incorrect, and the problem was resolved.

## Related work

Over the past 20 years, the R&E network community has developed numerous monitoring portals similar to NetSage. These include:

- The *ESnet portal*, my.es.net [47], includes dashboards for SNMP and flow data, and breaks down data based on both links and DOE sites, but only works with US DOE sites.
- The *GÉANT Tools Portal* [26] is a collection of public and restricted tools to view a wide variety of network monitoring data, including from Cacti and Looking Glass [41].
- The *Gloriad Portal* for the Gloriad network [13], active from 2006 to 2016, included SNMP and some flow data for a small subset of international R&E links, and also had a prototype of the Science Registry.
- *MONIT*, CERN’s monitoring portal MONIT for the Worldwide LHC Computing Grid (WLCG) [5, 46], provides tools for monitoring hosts and services in the CERN Data Centre and associated Computing Facilities, as well as experiment activities on the LHC Grid such as data transfers, job execution and site availability.
- *WorldView* [28], developed by IU Global NOC, uses SNMP data to show a map for many R&E networks worldwide.

Each of these portals were developed for use by a network operations center to provide a view for a single network provider or Science Experiment. NetSage is unique in that it was developed for a broader set of users over multiple networks, and to be able to analyze network performance related to data integrated from multiple networks and resources. NetSage Dashboards are designed primarily to understand performance issues and performance degradation, not outages.

In addition to the R&E community portals, there are a number of more general network measurement and monitoring tools developed by commercial (or semi-commercial) groups, again also to primarily support network operations center staff for a single network. The most relevant of these include:

- *Argus* [3] is a set of open source tools to collect and analyze flow data.
- *Cacti* [7] is open and supports SNMP out of the box but requires extensions for other data sources. The visualization options are also limited to the default set of graphs, unlike the library of plugins available to NetSage.
- *Deep Field* [17], primarily known in the R&E networking space for its use by Internet2, it uses flow data but has strict login and access control.
- *Elastiflow* [21] is similar to NetSage in approach, but not open source or privacy aware and is deployed only in a limited setting.
- *InMon* [36] is primarily focused on sFlow supported devices and does not support the range of protocols found in NetSage.
- *Kentik* [38] has several similar Dashboards to NetSage, but is deployed on a per-resource set approach, is not open, nor built for collecting network data across multiple organizations.
- *Nagios* [48] is an alarming service designed to do basic evaluation of data sets and notify operators. It is not designed to store time-series data at the scale of the network metrics collected by NetSage and assumes those are managed externally.
- *NFSEN* [73] is tool for displaying line graphs of Netflow data. Its data source and visualization options are much more narrowly focused than NetSage.
- *ntop* [74] is an open source set of tools that collect network metrics such as Netflow and SNMP data. The lack of perfSONAR support and Scientific metadata limit its ability to meet all the NetSage use cases.
- *SolarWinds* [83] is a commercial system for monitoring many of the same statistics as NetSage but is not open nor built for collecting network data across multiple organizations.

The NetSage Framework was influenced by, or leverages prior work from, some of these portals, such as the flow analysis capabilities of the ESnet portal and the Science Project database used in the now-defunct Gloriad Portal. None of these include all of the features supported by the NetSage Framework, for example, the ability to identify a science resource. Only Kentik and SolarWinds use both flow data and perfSONAR data, similar to the NetSage Framework, but neither of these are open source. No other monitoring system includes data such as Tstat for archival resources. In addition, none of them were created to openly share the level of detail that NetSage does to the general public, and across multiple networks and resources. A summary of all these tools is shown in Table 2. The most common feature supported by these tools that is not included in the NetSage Framework is alerting, which is planned as part of the next year’s development cycle.

**Table 2:**
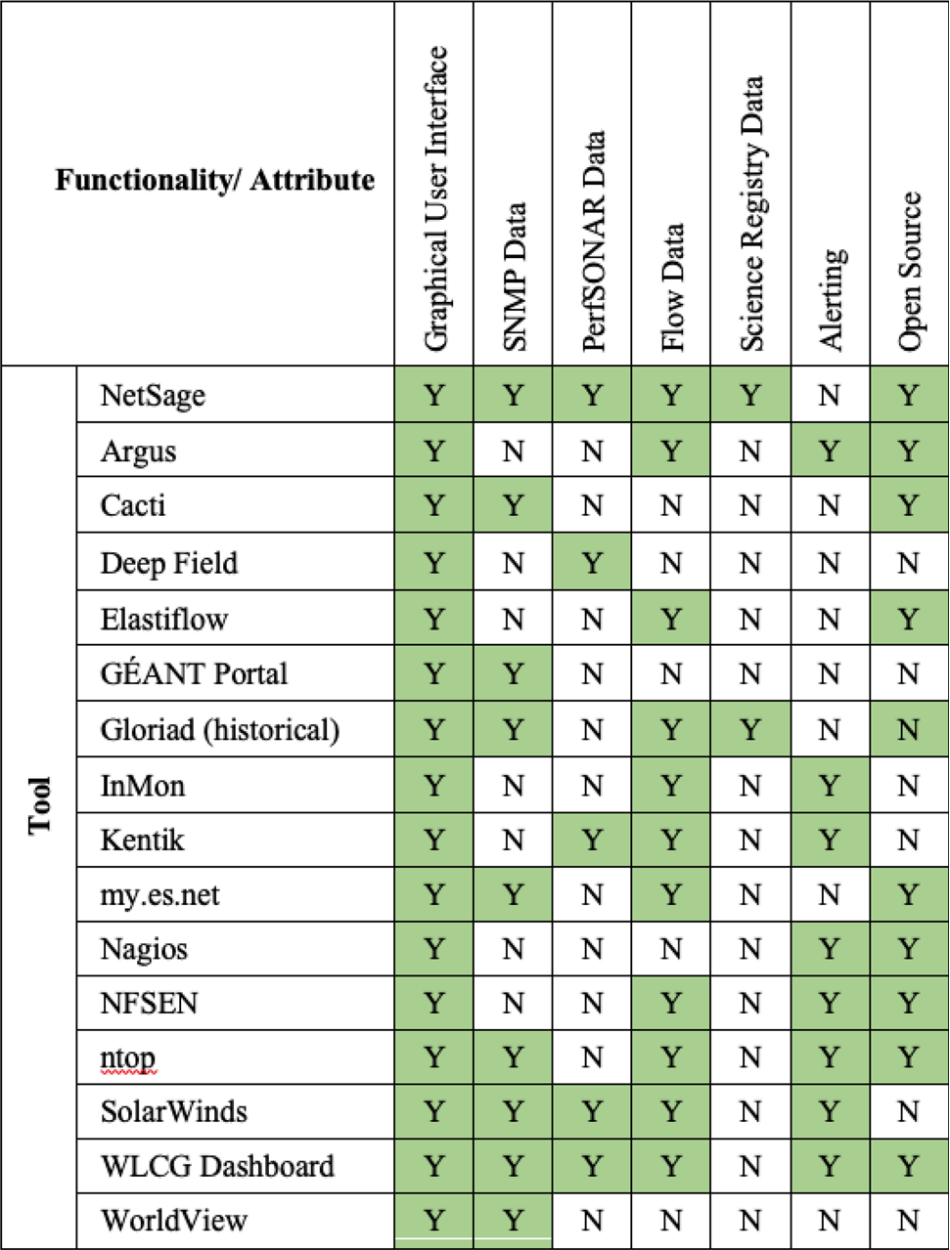
Comparison of related work

## Conclusion and future work

In this paper, we have described the NetSage design methodology and architecture as well as a broad set of use cases and discoveries. Within the space of R&E networks, we believe it is the most comprehensive open source approach to date that enables insight into underlying resource behaviors, and as such, differs significantly from other approaches which have been developed for NOC use only. NetSage enables insights across networks and resources by a broad set of end users, and each performance Dashboard is specifically developed to respond to a stakeholder question.

Future work will continue to be driven by stakeholder requests using our design methodology. In the short term we are developing additional visualizations as requested. For example, to identify the largest flows with the poorest performance among a set of resources, something no other monitoring system described above is capable of at this time. We continue to add more data to the Science Registry to be able to better reveal network use patterns of scientific applications, and to adapt the Project Dashboards in response to researcher needs. Longer term work includes adding in alarms and alerts and exploring adaptations needed to use a NetSage Deployment in a campus environment.
